# Deep-level phylogeny of Cicadomorpha inferred from mitochondrial genomes sequenced by NGS

**DOI:** 10.1038/s41598-017-11132-0

**Published:** 2017-09-05

**Authors:** Nan Song, Wanzhi Cai, Hu Li

**Affiliations:** 1 College of Plant Protection, Henan Agricultural University, Zhengzhou, 450002 China; 2 Department of Entomology, China Agricultural University, Beijing, 100094 China

## Abstract

Recent development and advancement of next-generation sequencing (NGS) technologies have enabled the determination of mitochondrial genome (mitogenome) at extremely efficiency. In this study, complete or partial mitogenomes for 19 cicadomorphan species and six fulgoroid species were reconstructed by using the method of high-throughput sequencing from pooled DNA samples. Annotation analyses showed that the mitogenomes obtained have the typical insect mitogenomic content and structure. Combined with the existing hemipteran mitogenomes, a series of datasets with all 37 mitochondrial genes (up to 14,381 nt total) under different coding schemes were compiled to test previous hypotheses of deep-level phylogeny of Cicadomorpha. Thirty-seven species representing Cicadomorpha constituted the ingroup. A taxon sampling with nine species from Fulgoroidea and six from Heteroptera comprised the outgroup. The phylogenetic reconstructions congruently recovered the monophyly of each superfamily within Cicadomorpha. Furthermore, the hypothesis (Membracoidea + (Cicadoidea + Cercopoidea)) was strongly supported under the heterogeneous CAT model.

## Introduction

The Cicadomorpha are generally regarded as an infraorder of Hemiptera. This insect group includes three superfamilies: Membracoidea (leafhoppers and treehoppers), Cicadoidea (cicadas) and Cercopoidea (spittlebugs and froghoppers), with approximately 30,000 described species^1^. The monophyly of the entire Cicadomorpha and of each superfamily has never been questioned based on morphological characters and/or molecular evidence. Internal relationships within Cicadomorpha have been addressed by several studies^2–11^, which attempted to assess higher hemipteran or auchenorrhynchan phylogeny and thus included representatives of superfamilies of Cicadomorpha. In particular, the higher-level relationships within Cicadomorpha were definitively resolved by the multi-locus, quantitative analyses of Cryan (2005)^10^ and Cryan & Urban (2012)^11^. Currently, most Hemiptera/Auchenorrhyncha systematists already agree that the arrangement of (Membracoidea + (Cicadoidea + Cercopoidea)) is the major relationship within Cicadomorpha.

Leston *et al*. (1954)^12^ proposed the infraorder Cimicomorpha. After that, Evans (1963) recovered a sister-group relationship between Cicadoidea and Membracoidea based mainly on the head morphological characters^13^. Hamilton (1981)^2^ placed Cicadoidea as an ancient lineage within Cicadomorpha and as a sister group of (Cercopoidea + Membracoidea), also based on the evidence from head morphology. In the subsequent molecular analyses based on 18*S rDNA* data^4, 5^, the hypothesis of (Cicadoidea + (Cercopoidea + Membracoidea)) was further supported. However, employing the similar 18*S rDNA* sequences, Campbell *et al*. (1995)^6^ recovered Cicadoidea and Cercopoidea as a clade, collectively sister to Membracoidea. The hypothesis of (Membracoidea + (Cicadoidea + Cercopoidea)) was supported by several morphology-based researches^9, 14–16^. Fossil evidence also supported the grouping of Cicadoidea with Cercopoidea^17, 18^. In the study by Cryan (2005)^10^, the same inter-superfamily relationship within Cicadomorpha as those in Campbell *et al*. (1995)^6^ was supported by the combination analyses of three nuclear markers (i.e. 18*S rDNA*, 28*S rDNA* and *histone 3*). Furthermore, the topological arrangement of (Membracoidea + (Cicadoidea + Cercopoidea)) was reinforced by expanding data from seven gene regions of nuclear and mitochondrial markers^11^. Therefore, the phylogenetic hypothesis of (Membracoidea + (Cicadoidea + Cercopiodea)) has been overwhelmingly supported by recent contributions as described above. This study aimed to take a phylogenomic approach based on additional mitochondrial genome (mitogenome) data to test relationships among superfamilies within Cicadomorpha.

Mitogenome is one of the most extensively utilized marker in phylogenetic studies of insects^19^. This class of organelle genome usually has high copy numbers^13^, for example, each human cell contains between 10^3^ and 10^4^ copies of the mitogenome^20^. This characteristic makes mtDNA easy to be determined. Complete mitogenome contains 37 mitochondrial genes, which are known to harbor higher substitution rates^21^. They are considered to be well-suited for resolving phylogenies at different taxonomic levels^22–29^. At present, compared with the whole genome data, mitogenome sequencing allows for much larger scale sampling. Especially in recent years, the advent of next-generation sequencing (NGS) techniques have revolutionized the ease with which mitogenome data can be obtained time- and also cost-effectively for large taxon sampling of insects^26, 30–36^.

In the following study, we determined 25 hemipteran insect mitogenome sequences including 19 cicadomorphan species and six fulgoroid species by using an approach of next-generation sequencing from mixed DNA samples. In addition, we combined with the published hemipteran mitogenome sequences to reconstruct the deep-level phylogeny of Cicadomorpha, with the goal of investigating relationships among superfamilies and families of this group.

## Materials and Methods

### Ethics Statement

No specific permits were required for the insect specimens collected for this study in China. These specimens were collected in the suburban fields of Xinyang, China. The field studies did not involve endangered or protected species. All sequenced insects are common species in China, and are not included in the “List of Protected Animals in China”.

### Taxon sampling

A total of 25 taxa were selected for mitogenome sequencing, with emphasis on the superfamily relationships within Cicadomorpha and the possible sister group lineage of Cicadomorpha (i.e. Fulgoroidea)^10^. Specimen identification were conducted by checking adult morphological characters, and by blasting in online identification tool of BOLD systems (Barcode Of Life Database: http://www.boldsystems.org - Identification section) and by the standard nucleotide BLAST in NCBI. The primary specimen materials can be accessed by the Entomological Museum of Henan Agricultural University. The detailed classification information and voucher numbers of species sequenced in this study are listed in Table S1.

In addition, we included all the current available mitogenomes of Membracoidea, Cicadoidea, Cercopoidea and Fulgoroidea in the GenBank (up to September 2016). In total, the ingroup included 37 taxa from Cicadomorpha: 17 species representing Membracoidea, eight species representing Cicadoidea, and 12 species representing Cercopoidea.

Outgroup choice included a taxon sampling based on the results of recent molecular studies^5, 6, 10, 11^. Nine species representing the superfamily of Fulgoroidea served as the close outgroups. Six species representing Heteroptera were selected as the relatively distant outgroups. The complete list of taxa included in this study is given in Table S1.

### DNA extraction

Total genomic DNA was isolated from each 95–100% ethanol preserved specimen individually using the TIANamp Micro DNA Kit (TIANGEN BIOTECH CO., LTD) following the manufacturer’s protocol. DNA concentration was measured by Nucleic acid protein analyzer (QUAWELL TECHNOLOGY INC.).

### Mitogenome reconstruction

The assembly strategy of complete mitogenome is largely identical to that of Gillett *et al*. (2014)^33^. The minor differences lied in the universal primers designed to amplify bait sequences as those in Song *et al*. (2016)^37^. Additionally, the Illumina HiSeq X Ten sequencing platform was utilized in the present study. Similar amounts of genomic DNA for each individual were pooled to improve the sequencing efficiency for every species. The amount of pooled DNA was quantified at 1.5 μg. Moreover, species with phylogenetically distant relations were mixed in a library to avoid the chimera formation. Totally, three libraries were constructed using Illumina TruSeq^TM^ DNA Sample Prep Kit (Illumina, San Diego, CA, USA). Genomic DNA were fragmented with a Covaris sonicator to an average insert size of 350 bp. The subsequent de novo genome sequencing was conducted on a single lane of Illumina HiSeq X Ten by Beijing Novogene Bioinformatics Technology Co., Ltd (China). Approximately 10 Gb paired-end reads of 150 bp length were generated for each library. FastQC^38^ was used for quality control of raw sequence data to remove reads containing adapters and ploy-N, or low quality reads from the raw data. All the downstream analyses were based on clean data of high quality (avg. Q20 > 90%, and avg. Q30 > 85%). No less than 8 Gb high-quality reads for each library were used in de novo assembly using IDBA-UD v. 1.1.1^39^, with default settings.

The information of bait sequences blasted against each mitochondrial contig determined are presented in Table S2. Only the assembled contigs to which at least one Sanger sequence could be matched with certainty were retained for further analysis. Mapping to identified mitochondrial contigs were performed using BWA v 0.7.13^40^ under default parameters. Mapping statistics were obtained with Qualimap^41^ and Tablet^42^, in order to check the quality of the assembled contigs.

The preliminary annotation for the contigs identified by bait sequences were conducted using MITOS^43^, with default settings and the invertebrate genetic code for mitochondria. The resultant gene boundaries were further checked and corrected by alignment against 20 published mitogenome sequences from Cicadomorpha and Fulgoroidea (see details in Table S1). Furthermore, hand alignment checking was also conducted by blasting each predicted gene for every mitogenome against GenBank data in Web BLAST (https://blast.ncbi.nlm.nih.gov/Blast.cgi), to ensure the identity of the mitochondrial genes identified.

### Sequence alignment

The nucleotide sequences of each protein-coding gene were aligned based on codons using the invertebrate mitochondrial genetic code in the Perl script TransAlign^44^. Each of tRNA and rRNA genes was aligned using MAFFT (version 7)^45^ under iterative refinement method incorporating the most accurate local (E-INS-i) pairwise alignment information. Alignments were checked in MEGA 6^46^. Gaps were striped by Gap Strip/Squeeze v2.1.0 with 40% Gap tolerance (http://www.hiv.lanl.gov/content/sequence/GAPSTREEZE/gap.html). Finally, all alignments were concatenated to construct two matrices using FASconCAT_v1.0^47^, one including RNA genes (i.e. PCG), and one excluding RNA genes (i.e. PCGRNA).

A part from two datasets mentioned above, a series of datasets were compiled by the following methods to test the influences of recoding schemes on the phylogenetic estimate: (1) PCG_AA: translating nucleotides of protein-coding genes into amino acid sequences; (2) PCGDegen: 13 protein-coding genes were re-coded by degenerating all sites including synonymous substitutions to IUPAC ambiguity codes through Degen v1.4 (Degen-code)^48, 49^; (3) PCGDegenRNA: 13 protein-coding genes with Degen-coding combined with 24 RNA genes. Additionally, the datasets of PCG and PCGRNA were masked by using Aliscore version 2.0^50, 51^, combined with Alicut version 2.0^50, 51^. Finally, to investigate the potential long-branch effect on the tree topology, a series of reduced datasets (i.e. 43 taxa datasets without the outgroup Fulgoroidea) were created to further phylogenetic analyses.

Sequence saturation in the combined protein-coding genes and RNA genes were assessed using the index of substitution saturation (*Iss*)^52^ as implemented in the DAMBE 5^53^, respectively. Estimates of nonsynonymous (*dN*) and synonymous (*dS*) substitution rates of concatenated protein-coding genes were obtained by the method of Yang and Nielsen (2000)^54^ using the program yn00 as implemented in PAML 4.9^55^. The One-way Analysis of Variance (ANOVA) is performed in Excel 2016.

### Phylogenetic analyses

Phylogenetic reconstructions were based on the mitogenome sequences of the full datasets with 52 taxa and the reduced datasets with 43 taxa under maximum likelihood (ML) and Bayesian inference (BI).

Prior to ML analyses, PartitionFinder^56^ was employed to infer the optimal partitioning strategy. Simultaneously, the Baysian Information Criterion (BIC) were used to choose the best models for the combined nucleotide and amino acid datasets under a greedy search with RAxML^57, 58^. The data blocks were defined by gene types (each of 13 PCGs, 22 tRNAs and two rRNAs) and by codon positions (each of three codon positions for PCGs), totally 63 independent blocks were employed for the datasets of 52taxa_PCGRNA, 52taxa_PCGDegenRNA, 43taxa_PCGRNA, and 43taxa_PCGDegenRNA. The partition schemes and best-fit models selected for each dataset are provided in Table S3. Gene types and codon positions cannot be distinguished when data were masked, thus no partition analyses were applied to these datasets (i.e. Alicut-52taxa_PCG, Alicut-52taxa_PCGRNA, Alicut-43taxa_PCG, and Alicut-43taxa_PCGRNA).

ML analyses were conducted using IQ-TREE^59^ as implemented on the multicore version of IQ-TREE 1.5.5. The partition schemes and best-fit models selected by PartitionFinder were applied to corresponding dataset. Branch support was estimated using Ultrafast option for bootstrap analysis, with 1000 replicates. The detailed commands are as following: iqtree-omp -s dataset.nex -st DNA (or AA) -bb 1000 -alrt 1000.

Bayesian analyses were performed using a parallel version of PhyloBayes (pb_mpi1.5a)^60, 61^ as implemented on a HP server with twenty-four CPU and 320 G memory. For all Phylobayes analyses, two independent runs were performed, and started from random topology, respectively. Each run implemented two differentially heated chains, with at least 30,000 cycles. The CAT-GTR model was used for nucleotide analyses, while the CAT model for amino acids. Convergence was monitored using bpcomp (“maxdiff” value < 0.1) and tracecomp (minimum effective size > 100). A 25% burn-in was applied after checking for stationarity, and a consensus tree was calculated.

For each phylogenetic analysis, we utilized FigTree v1.4.3^62^ to visualize the consensus tree and the corresponding branch lengths. The one-way ANOVA analyses for branch lengths of major groups are performed in Excel 2016. The bootstrap supports (BS) of ≥75 and posterior probabilities (PP) of ≥0.95 were considered to be credible support values for tree nodes. All sequence alignment files and tree files built in this article are available in the Treebase: http://purl.org/phylo/treebase/phylows/study/TB2:S19876.

### Hypothesis testing

To test the statistical significance of alternative hypotheses of Cicadomorpha, we compared all possible relationships among three superfamilies, namely (Membracoidea + (Cicadoidea + Cercopoidea)), (Cicadoidea + (Membracoidea + Cercopoidea)), and (Cercopoidea + (Membracoidea + Cicadoidea)). The topology tests were conducted using the datasets with the full 52 taxa (i.e. 52taxa_PCG, 52taxa_PCGDegen, 52taxa_PCG_AA, 52taxa_PCGRNA, 52taxa_PCGDegenRNA, Alicut_52taxa_PCG, and Alicut_52taxa_PCGRNA). The site-log-likelihood values were calculated under the GTR + I + G model for nucleotides and the MtREV + I + G model for amino acids using TREE-PUZZLE 5.3^63^ (the command-line option: -wsl). The obtained values were used as input for the software CONSEL^64^. Constraint likelihood trees were constructed on the basis of dataset of 52taxa_PCGRNA with RAxML^57, 58^ using the model GTRGAMMA and using partitions selected by PartitionFinder. Three competing hypotheses were statistically tested among each other by AU^65^, KH^66^, SH^67^, WKH and WSH.

## Results

### Assembly of mitogenomes

**Figure 1:**
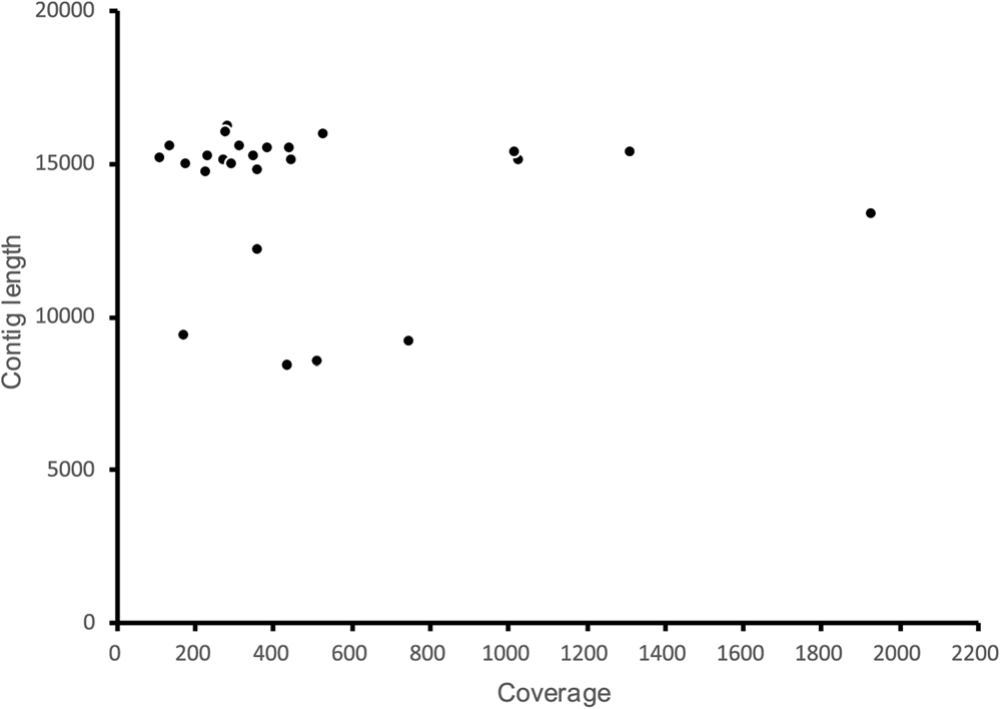
The graph of mean sequencing coverage versus contig length for 25 identified mitogenomic assemblies.

Twenty-five hemipteran insect mitogenomes determined were identified from the individual contigs by bait sequences. No chimeric formation was found when we inspected the base coverage along each mitogenome with the software Tablet^42^. Because every site in each mitochondrial contig corresponded to an identical nucleotide. The statistics from the program BWA^40^ showed that read coverage for all gene regions in each contig was no less than 30-fold. Mean coverage for every mitogenome varied between 103-fold and 1,922-fold. The average depth of coverage was 475-fold, with 17 contigs ranging from 220-fold to 742-fold coverage and four contigs at >1,000-fold coverage (Fig. 1). Although with the higher sequence coverage, complete mitogenomes (including the full 37 mitochondrial genes and the entire control region) were identified for seven insect species. The remainder were nearly complete (12 mitogenomes including the full 37 mitochondrial genes and the partial control region) or partial mitogenomes (six mitogeonomes including 20–35 mitochondrial genes and no partial control region). The lengths of the mitogenome sequences ranged from 8,455 nt to 16,226 nt, of which 17 mitogenomes had sequences’ length more than 15,033 nt, four ones ranged from 12,231 nt to 14,803 nt, and the rest had sequences < 10,000 nt. For the incomplete mitogenomes, the missing segments were mainly located adjacent to the putative control region. The complete or nearly complete mitogenomes have the consistent gene content and organization with other published auchenorrhynchan insect mitogenomes. All new mitogenome sequences have been deposited in GenBank (accession numbers are presented in Table S1).

The results of the substitution saturation tests based on the concatenated protein-coding genes with 52 taxa showed that the values of substitution saturation index (*Iss*) for the first and/or second codons and all sites of PCG were significantly smaller than the critical values (*Iss.cSym* or *Iss.cAsym*). However, the *Iss* values of the third codon positions were larger than the *Iss.cSym* and *Iss.cAsym* (Table 1). This result indicated that the third codon positions might have a negative impact on phylogenetic analysis. Removal of the long-branched outgroups (i.e. Fulgoromorpha) did not significantly reduced the saturation degree. There still showed saturation in the third codon positions and in the RNA genes only (Table 1). From the point of view of the lower *Iss* values, data masking reduced the degree of saturation.

**Table 1:** Saturation test performed by DAMBE.

Gene regions	NumOTU	*Iss*	*Iss.cSym*	P	*Iss.cAsym*	P
52taxa_PCG1	32	0.529	0.808	0.0000	0.553	0.0303
52taxa_PCG2	32	0.398	0.808	0.0000	0.553	0.0000
52taxa_PCG1 + 2	32	0.458	0.814	0.0000	0.570	0.0000
52taxa_PCG3	32	**0.821**	**0.808**	0.1088	**0.553**	0.0000
52taxa_PCG123	32	0.558	0.818	0.0000	0.572	0.0000
52taxa_Alicut-PCG	32	0.449	0.815	0.0000	0.571	0.0000
52taxa_RNA	32	**0.705**	0.807	0.0000	**0.550**	0.0000
43taxa_PCG1	32	0.487	0.808	0.0000	0.553	0.0000
43taxa_PCG2	32	0.351	0.808	0.0000	0.553	0.0000
43taxa_PCG1 + 2	32	0.416	0.814	0.0000	0.570	0.0000
43taxa_PCG3	32	**0.806**	0.808	0.8142	**0.553**	0.0000
43taxa_PCG123	32	0.524	0.818	0.0000	0.572	0.0000
43taxa_Alicut-PCG	32	0.394	0.815	0.0000	0.571	0.0000
43taxa_RNA	32	**0.658**	0.807	0.0000	**0.550**	0.0000

Note: The occurrences of saturation are indicated in bold.

To explore the correlation between sequence evolution rate and tree topology, we compared the nonsynonymous (*dN*) and synonymous (*dS*) evolutionary rates for each sequence pair within five groups base on the concatenated protein-coding genes (Table 2). The one-way ANOVA analysis revealed incongruence in the *dN* values between groups (*P* = 0.0429). This result was mainly due to the lower *dN* values of Cercopoidea. When we omitted the Cercopoidea to rerun one-way ANOVA analysis, there was no significant difference across all remaining lineages. There was no significant difference between major groups in the *dS* values (*P* = 0.0539) or in the *dN*/*dS* values (*P* = 0.0697).

**Table 2:** Estimation of nonsynonymous (*dN*) and synonymous (*dS*) substitution rates by yn00 implemented in PAML.

**Group**	**Higher taxa**	**Species**	***dN***	***dS***	***dN/dS***
1	Cercopoidea	*Abidama producta*	0.1875	4.5129	0.0416
1	Cercopoidea	*Aeneolamia contigua*	0.1869	4.5579	0.0410
1	Cercopoidea	*Aphrophora alni*	0.1942	4.4320	0.0438
1	Cercopoidea	*Aphrophora* sp.	0.1893	4.6337	0.0409
1	Cercopoidea	*Callitettix biformis*	0.1900	4.6523	0.0408
1	Cercopoidea	*Callitettix braconoides*	0.1897	4.5338	0.0419
1	Cercopoidea	*Callitettix* sp.	0.1900	4.5334	0.0419
1	Cercopoidea	*Callitettix versicolor*	0.1910	4.5573	0.0419
1	Cercopoidea	*Cosmoscarta bispecularis*	0.2131	4.7267	0.0451
1	Cercopoidea	*Paphnutius ruficeps*	0.2087	4.5719	0.0456
1	Cercopoidea	*Peuceptyelus minutus*	0.2401	4.6554	0.0516
1	Cercopoidea	*Philaenus spumarius*	0.2197	4.6640	0.0471
2	Cicadoidea	*Gaeana maculata*	0.2100	4.6076	0.0456
2	Cicadoidea	*Magicicada tredecim*	0.2998	4.6179	0.0649
2	Cicadoidea	*Meimuna opalifera*	0.2605	4.5730	0.0570
2	Cicadoidea	*Platypleura kaempferi*	0.2114	4.7246	0.0447
2	Cicadoidea	*Pomponia linearis*	0.2039	4.6080	0.0442
2	Cicadoidea	*Tettigades auropilosa*	0.2475	4.6274	0.0535
2	Cicadoidea	*Tettigades ulnaria*	0.2247	4.7728	0.0471
2	Cicadoidea	*Diceroprocta semicincta*	0.1943	4.6352	0.0419
3	Fulgoroidea	*Lycorma delicatula*	0.2849	4.7153	0.0604
3	Fulgoroidea	*Lydda* sp.	0.2465	4.6355	0.0532
3	Fulgoroidea	*Nilaparvata* sp.	0.2265	4.8451	0.0467
3	Fulgoroidea	*Oliarus* sp.	0.2122	4.6527	0.0456
3	Fulgoroidea	*Pentastiridius* sp.	0.2056	4.6321	0.0444
3	Fulgoroidea	*Scolops* sp.	0.2186	4.6690	0.0468
3	Fulgoroidea	*Sivaloka damnosus*	0.2278	4.6292	0.0492
3	Fulgoroidea	*Sivaloka* sp.	0.2048	4.6000	0.0445
3	Fulgoroidea	*Sogatella furcifera*	0.2134	4.7127	0.0453
4	Heteroptera	*Diplonychus rusticus*	0.2104	4.6572	0.0452
4	Heteroptera	*Enithares tibialis*	0.2160	4.5846	0.0471
4	Heteroptera	*Hydaropsis longirostris*	0.2154	4.5687	0.0472
4	Heteroptera	*Lygus hesperus*	0.2475	4.6338	0.0534
4	Heteroptera	*Macroscytus gibbulus*	0.2578	4.6158	0.0558
4	Heteroptera	*Yemmalysus parallelus*	0.2134	4.7320	0.0451
5	Membracoidea	*Alobaldia tobae*	0.1929	4.4817	0.0430
5	Membracoidea	*Darthula hardwickii*	0.1927	4.5987	0.0419
5	Membracoidea	*Drabescoides nuchalis*	0.2143	4.5832	0.0468
5	Membracoidea	*Empoasca vitis*	0.2206	4.6509	0.0474
5	Membracoidea	*Exitianus indicus*	0.2110	4.6075	0.0458
5	Membracoidea	*Homalodisca vitripennis*	0.2207	4.6315	0.0477
5	Membracoidea	*Illinigina* sp.	0.2587	4.6322	0.0559
5	Membracoidea	*Leptobelus gazella*	0.2865	4.5969	0.0623
5	Membracoidea	*Nephotettix cincticeps*	0.2855	4.7262	0.0604
5	Membracoidea	*Norvellina* sp.	0.2093	4.6171	0.0453
5	Membracoidea	*Olidiana* sp.	0.2122	4.6698	0.0454
5	Membracoidea	*Orosius orientalis*	0.2226	4.6732	0.0476
5	Membracoidea	*Phlogotettix* sp.	0.2291	4.6404	0.0494
5	Membracoidea	*Tricentrus* sp.	0.2061	4.6091	0.0447
5	Membracoidea	*Tricentrus* sp.1	0.2344	4.6922	0.0500
5	Membracoidea	*Typhlocyba* sp.	0.2279	4.6335	0.0492
5	Membracoidea	*Yanocephalus yanonis*	0.2184	4.6406	0.0471

### Phylogenetic analyses

**Figure 2:**
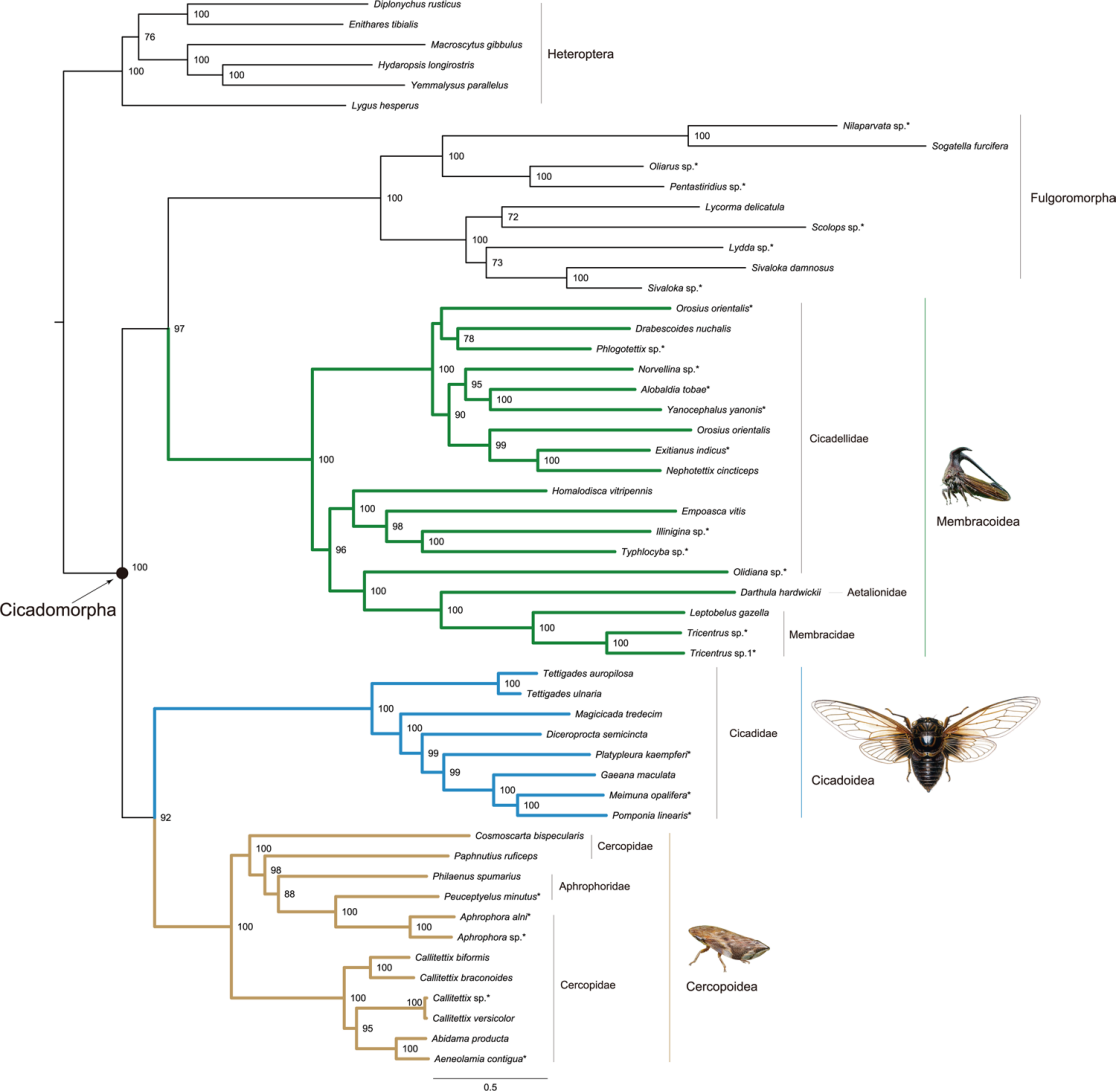
Maximum likelihood tree inferred from the dataset of 52taxa_PCGRNA using IQ-TREE under the partition schemes and best-fit models selected by PartitionFinder. Node numbers show bootstrap support values (≥70). Scale bar represents substitutions/site. Asterisks designate the species newly sequenced in this study. Other ML trees reconstructed in this paper can be available from the TreeBase: http://purl.org/phylo/treebase/phylows/study/TB2:S19876.

ML analyses based on the full taxa datasets (i.e. 52taxa_PCG, 52taxa_PCGDegen, 52taxa_PCGRNA, 52taxa_PCGDegenRNA, 52taxa_PCG_AA, Alicut_52taxa_PCG, and Alicut_52taxa_PCGRNA) consistently resulted in a paraphyletic Cicadomorpha, with respect to the nested position of Fulgoroidea (Fig. 2). Monophyly of each superfamily within Cicadomorpha was very strongly supported (BP = 100). The sister group relationship between Cicadoidea and Cercopoidea was favored with strong nodal support (BP ≥ 85) in all analyses. Table 3 provides the nodal supports and branch lengths for major lineages in each tree. Comparing the support values of deep nodes in each tree, we found that data treatment decreased the support for the inter-superfamily relationships. Specially, the dataset PCGDegenRNA showed the lowest support for the affinity of Cicadoidea with Cercopoidea. This suggested synonymous sites may provide information for the deep-level relations within Cicadomorpha. The one-way ANOVA analysis showed no significant incongruence in the branch lengths between major groups (*P* > 0.05) in the ML analyses. At the family level, the Membracidae were congruently supported as a monophyletic lineage (BP = 100). However, the Cicadellidae was recovered as a paraphyletic assemblage, owing to the close affinity of *Olidiana* sp. to the clade (Aetalionidae + Membracidae). Within Cercopoidea, both families Aphrophoridae and Cercopidae were supported as the non-monophyletic groups.

**Table 3:** Nodal supports and branch lengths for major lineages in each tree.

Dataset	Fulgoroidea		Cicadoidea		Cercopoidea		Membracoidea	
	NS	BL	NS	BL	NS	BL	NS	BL
Maximum likelihood analyses using IQ-TREE								
52taxa_PCG	100	5.14	100	3.33	100	2.59	100	4.06
52taxa_PCGDegen	100	1.01	100	0.59	100	0.50	100	0.79
52taxa_PCG_AA	100	1.61	100	0.91	100	0.78	100	1.18
52taxa_PCGRNA	100	3.78	100	2.37	100	1.78	100	2.95
52taxa_PCGDegenRNA	100	1.00	100	0.57	100	0.46	100	0.78
Alicut_52taxa_PCG	100	0.66	100	0.48	100	0.42	100	0.52
Alicut_52taxa_PCGRNA	100	0.64	100	0.40	100	0.34	100	0.50
43taxa_PCG	—	—	100	3.82	100	3.05	100	4.83
43taxa_PCGDegen	—	—	100	0.52	100	0.46	100	0.75
43taxa_PCG_AA	—	—	100	0.91	100	0.73	100	1.23
43taxa_PCGRNA	—	—	100	2.36	100	2.05	100	3.45
43taxa_PCGDegenRNA	—	—	100	0.59	100	0.55	100	0.95
Alicut_43taxa_PCG	—	—	100	0.44	100	0.38	100	0.50
Alicut_43taxa_PCGRNA	—	—	100	0.39	100	0.32	100	0.47
Bayesian analyses using PhyloBayes								
52taxa_PCG	1.00	4.09	1.00	2.14	0.83	1.77	1.00	3.43
52taxa_PCGDegen	1.00	1.97	1.00	0.78	0.58	0.73	1.00	1.48
52taxa_PCG_AA	1.00	3.96	1.00	1.86	1.00	1.85	1.00	3.05
52taxa_PCGRNA	1.00	3.70	1.00	1.89	1.00	1.47	1.00	3.15
52taxa_PCGDegenRNA	1.00	2.28	1.00	0.93	1.00	0.81	1.00	1.79
Alicut_52taxa_PCG	1.00	4.04	1.00	2.13	1.00	1.78	1.00	3.42
Alicut_52taxa_PCGRNA	1.00	4.05	1.00	1.59	1.00	1.43	1.00	2.98
43taxa_PCG	—	—	1.00	2.17	1.00	1.50	1.00	3.55
43taxa_PCGDegen	—	—	1.00	0.71	1.00	0.61	1.00	1.40
43taxa_PCG_AA	—	—	1.00	1.60	1.00	1.53	1.00	2.66
43taxa_PCGRNA	—	—	1.00	1.85	1.00	1.29	1.00	3.19
43taxa_PCGDegenRNA	—	—	1.00	0.93	1.00	0.68	1.00	1.88
Alicut_43taxa_PCG	—	—	1.00	2.32	1.00	1.71	1.00	3.52
Alicut_43taxa_PCGRNA	—	—	1.00	1.60	1.00	1.36	1.00	3.43

Note: The branch lengths were calculated from the longest terminal taxon of each lineage to the common ancestor to the Heteroptera. “−” denotes the monophyletic lineage not to be recovered by the dataset. NS: nodal support; BL: branch length.

**Figure 3:**
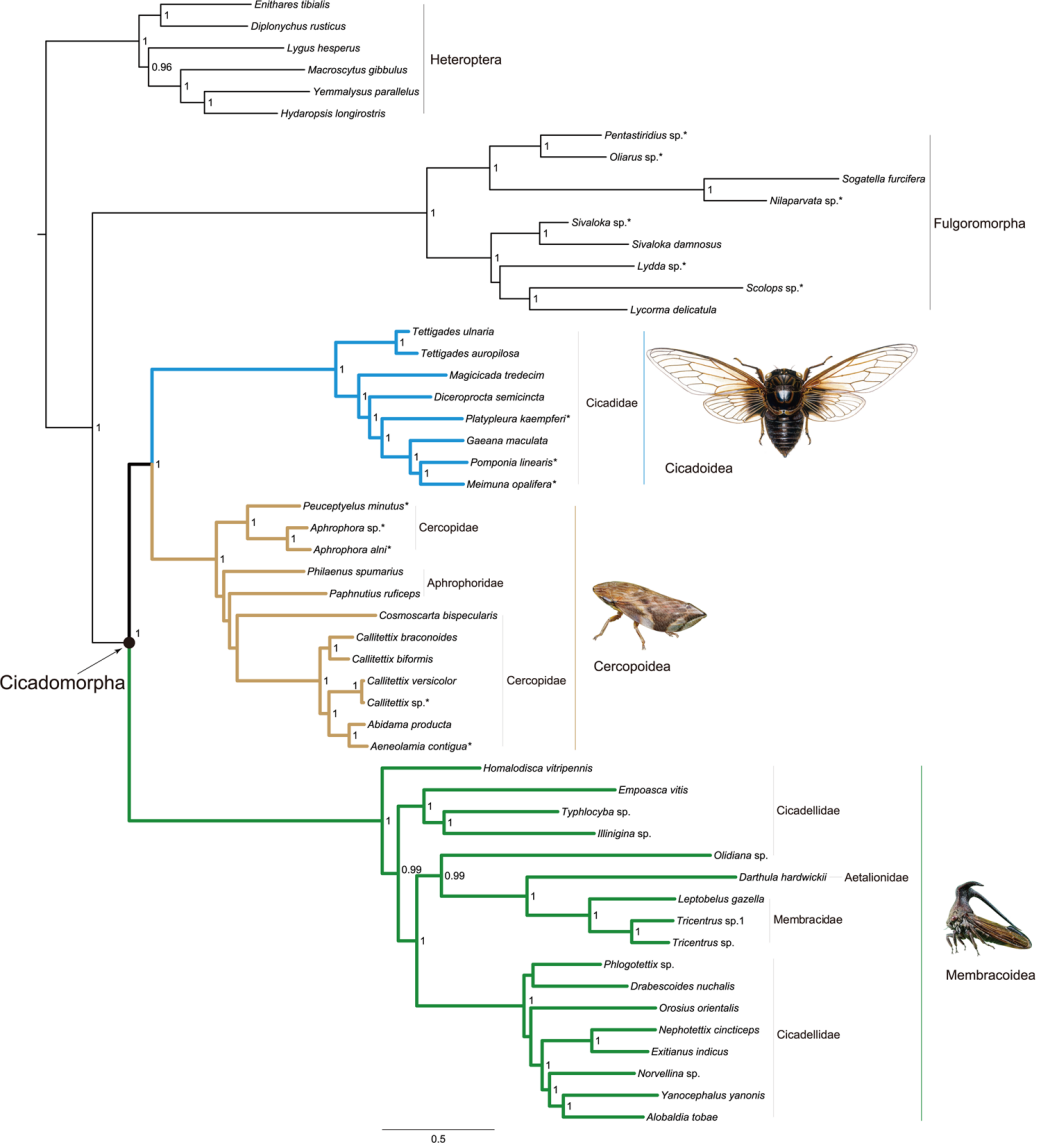
Bayesian tree inferred from the dataset of 52taxa_PCGRNA using PhyloBayes under the site-heterogeneous CAT-GTR model. Node numbers show poster probability values (≥0.95). Scale bar represents substitutions/site. Asterisks designate the species newly sequenced in this study. Other Bayesian trees reconstructed in this paper can be available from the TreeBase: http://purl.org/phylo/treebase/phylows/study/TB2:S19876.

Bayesian analyses based on the full taxa datasets under the heterogeneous model provided the distinct tree topology from those in the ML analyses. All Bayesian analyses recovered the monophyly of Cicadomorpha (Fig. 3), except for the analysis based on the 52taxa_PCG_AA. The Cicadoidea formed a sister group to the Cercopoidea, with posterior probability 1 in the analyses of 52taxa_PCGRNA, 52taxa_PCGDegenRNA, Alicut_52taxa_PCG, and Alicut_52taxa_PCGRNA. The Membracoidea was sister to the clade (Cicadoidea + Cercopoidea). The datasets with recoding schemes displayed the weaker power in resolving the deep-level phylogeny of Cicadomorpha. The branching pattern of (Membracoidea + (Cicadoidea + Cercopoidea)) was not retrieved in the Bayesian trees from PCGDegen and PCG_AA. The dataset PCGDegen showed the lowest nodal support for the sister group relation of Cicadoidea + Cercopoidea. The one-way ANOVA analysis revealed significant incongruence in the branch lengths between major groups (*P* < 0.05) in the Bayesian analyses. When removing the Fulgoroidea and Membracoidea to rerun the ANOVA analysis, there was no difference between Cicadoidea and Cercopoidea. This result demonstrated that the potential long-branch effect was introduced by the Fulgoroidea and Membracoidea.

### Taxon deletion experiment

The taxon deletion experiments were conducted to test for the effect of long-branch attraction. For that, a series of ML analyses were rerun based on the 43 taxa datasets. As a result, all ML analyses with 43 taxa datasets yielded strong evidence for a monophyletic Cicadomorpha (BP = 100), with an inter-superfamily relationship of (Membracoidea + (Cicadoidea + Cercopoidea)). The deep nodes defining superfamily relationships received strong bootstrap support values (BP ≥ 78).

Similarly, the majority of Bayesian analyses based on the 43 taxa datasets reproduced the superfamily relationships within Cicadomorpha as ML analyses based on the 43 taxa datasets. With the exception of 43taxa_PCGDegenRNA, all 43 taxa datasets showed Cicadoidea as sister group to Cercopoidea, and together they were sister to Membracoidea. In the Bayesian tree of 43taxa_PCGDegenRNA, Membracoidea was placed as sister group to Cercopoidea, while Cicadoidea was recovered as an independent lineage within Cicadomorpha.

### Hypothesis testing

Various tests consistently supported the arrangement of (Membracoidea + (Cicadoidea + Cercopoidea)) as the best tree topology, regardless of data coding schemes applied (Table 4). In contrast, all tests but for those on the ALICUT_52taxa_PCGRNA significantly rejected the hypothesis of (Cicadoidea + (Membracoidea + Cercopoidea)). Additionally, the comparison of topologies clearly rejected the alternative hypothesis of (Cercopoidea + (Membracoidea + Cicadoidea)).

**Table 4:** Tree topology testing.

**Datasets**	**Hypothesis**	**−Ln likelihood**	**AU**	**KH**	**SH**	**WKH**	**WSH**
52taxa_PCG	Mem + (Cic + Cer)	−327098.5	1.000	1.000	1.000	1.000	1.000
	Cic + (Cer + Mem)	−327209.6	**0.000**	**0.000**	**0.002**	**0.000**	**0.001**
	Cer + (Cic + Mem)	−327199.5	**0.000**	**0.000**	**0.001**	**0.000**	**0.000**
52taxa_PCGDegen	Mem + (Cic + Cer)	−135979.4	0.997	0.997	1.000	0.992	1.000
	Cic + (Cer + Mem)	−136046.9	**0.007**	**0.008**	**0.019**	**0.008**	**0.012**
	Cer + (Cic + Mem)	−136039.7	**0.004**	**0.003**	**0.028**	**0.003**	**0.006**
52taxa_PCG_AA	Mem + (Cic + Cer)	−170376.6	0.998	0.998	1.000	0.998	1.000
	Cic + (Cer + Mem)	−170459.9	**0.002**	**0.002**	**0.007**	**0.002**	**0.003**
	Cer + (Cic + Mem)	−170477.7	**0.000**	**0.000**	**0.002**	**0.000**	**0.000**
52taxa_PCGRNA	Mem + (Cic + Cer)	−412235.2	1.000	0.998	1.000	0.998	1.000
	Cic + (Cer + Mem)	−412338.4	**0.000**	**0.002**	**0.006**	**0.002**	**0.002**
	Cer + (Cic + Mem)	−412353.9	**0.000**	**0.000**	**0.001**	**0.000**	**0.000**
52taxa_PCGDegenRNA	Mem + (Cic + Cer)	−219796.2	0.994	0.989	1.000	0.989	1.000
	Cic + (Cer + Mem)	−219863.1	**0.009**	**0.011**	**0.038**	**0.011**	**0.019**
	Cer + (Cic + Mem)	−219883.2	**0.001**	**0.001**	**0.006**	**0.001**	**0.001**
ALICUT_52taxa_PCG	Mem + (Cic + Cer)	−232954.9	0.980	0.966	0.999	0.966	0.998
	Cic + (Cer + Mem)	−233040.1	**0.038**	**0.034**	0.084	**0.034**	0.058
	Cer + (Cic + Mem)	−233064.7	**0.003**	**0.004**	**0.026**	**0.004**	**0.008**
ALICUT_52taxa_PCGRNA	Mem + (Cic + Cer)	−281229.9	0.897	0.890	0.982	0.890	0.979
	Cic + (Cer + Mem)	−281292.7	0.112	0.110	0.206	0.110	0.171
	Cer + (Cic + Mem)	−281387.9	**0.001**	**0.000**	**0.006**	**0.000**	**0.001**

Note: Bold indicates the values of *p* < 0.05. Mem: Membracoidea, Cic: Cicadoidea, Cer: Cercopoidea.

## Discussion

### Efficiency of reconstructing complete mitogenomes

The present study demonstrates the utility of next-generation sequencing of mixed DNA samples for reconstructing hemipteran mitogenomes. This method shows a great improvement in the efficiency of achieving a large number of mitogenome data, compared with the traditional Sanger sequencing via primer walking. We used this method to successfully reconstruct 19 cicadomorphan insect mitogenomes and six fulgoroid insect mitogenomes. The number of mitogenome with full-length sequence is relatively small, with only seven ones. For the remaining nearly complete or partial mitogenomes, the missing segments are mainly located in the control region and/or including the adjacent regions. The low capture specificity of the control region could be attributed to the following reasons: 1) the complex nucleotide motifs found in this region (e.g. A + T-rich elements); 2) non-uniform read coverage along the mitogenome determined, especially with the significant drops occurred in the segments corresponding to the control region. Both factors may lead to the sequencing failure and lower assembly efficiency of this specific region. According to previous studies^33, 34, 68^, the current sequencing depth should be sufficient to cover the whole mitogenome. To some extent, more taxon sampling can be added to the pools to improve the sequencing efficiency. Therefore, in order to ensure the completeness of mitogenome, how to increase the sequencing depth for the particular regions of genome and how to assemble the complete mitogenome by developing more efficient assembler will be other important issues.

### Outgroup selection for phylogenetic analysis of Cicadomorpha

Outgroup choice is critical in deep phylogenetic studies^69^, because it determines the polarity of characters analyzed. Problematic tree rooting has caused conflict results in the previous studies on the phylogeny of Cicadomorpha^5, 6, 10^. Cryan (2005) discussed the issues on the selection of outgroup in the phylogenetic reconstruction of Cicadomorpha^10^. Despite uncertainties remain on the relationships between Cicadomorpha and their allies, Cryan (2005)^10^ thought that it was appropriate to utilize the Fulgoroidea as the outgroup in his study. Besides, he suggested that analysis of Cicadomorpha phylogeny should include representatives from Heteroptera as outgroups^10^. In the current study, we applied an outgroup taxon sample including Heteroptera and Fulgoroidea to the tree reconstruction of Cicadomorpha. Unfortunately, long branch lengths shared by outgroup Fulgoroidea and ingroup Membracoidea were found in the current mitogenome data (Table 3). Moreover, evolutionary rate analyses indicated the distinct values of nonsynonymous substitution across five major groups analyzed. These results made us to suspect that incongruence between analyses of two inference methods (i.e. ML analysis under homogeneous model and Bayesian analysis under heterogeneous model) from the full datasets might be the consequence of long-branch effect. Excluding long-branched outgroup Fulgoroidea resulted in a congruent result from both inference methods. Cicadomorpha was recovered as a monophyletic group, with the internal relationship of (Membracoidea + (Cicadoidea + Cercopoidea)). Therefore, the Heteroptera may be a more suitable outgroup choice to the Cicadomorpha phylogeny estimate on the current mitogenome data, due to the similar evolutionary rate shared by them.

### Deep-level phylogeny within Cicadomorpha

For the superfamily relationship of (Membracoidea + (Cicadoidea + Cercopoidea)) within Cicadomorpha, Boulard (1991a,b)^70, 71^ listed two character states supporting this hypothesis: (1) structures of the alimentary canal; (2) the larval behavior of applying excreted liquid. Liang & Fletcher (2002)^14^ proposed the common antennal features shared by Cicadoidea and Cercopoidea. Rakitov (2002)^15^ listed structural characters of brochosomes and proteinaceous particles secreted by glandular regions of the Malpighian tubules as synapomorphic for the grouping (Cicadoidea + Cercopoidea). In addition, fossil researches proved the close affinity of Cicadoidea with Cercopoidea^7, 17^. Recent molecular studies^10, 11^, based mainly on the nuclear gene fragments, also recovered a solid branch pattern of (Membracoidea + (Cicadoidea + Cercopoidea)). Results presented in the current study are the first molecular investigation to utilize mitogenome data only for reconstructing deep-level phylogeny of Cicadomorpha. The majority of our analyses recovered the topology of (Membracoidea + (Cicadoidea + Cercopoidea)), corroborating earlier studies with new data. Nevertheless, the causes for another recovery of (Cicadoidea + (Membracoidea + Cercopoidea)) by only one analysis based on 43taxa_PCGDegenRNA under PhyloBayes may be complex. It is possible that a combined effect of Degen-coding and additional RNA genes contributed to the latter tree structure.

Taken together, the deep-level phylogeny of Cicadomorpha was explored using mitogenome data, with various coding strategies and different algorithms. Our results validate the power of mitogenome for resolving the relationships at the superfamily level in Cicadomorpha. Although the number of mitogenomes available for Cicadomorpha has been almost doubled by this study, phylogenetic result is still preliminary for this megadiverse insect group. In particular, analysis of family level relationship within Cicadomorpha will require more taxon sampling. The related research project is being carried out by the authors.

## Electronic supplementary material

Supplementary files
