# Supplementary material for: Deep-level phylogeny of Cicadomorpha inferred from mitochondrial genomes sequenced by NGS
Source: Sci Rep. 2017 Sep 5;7:10429. doi: 10.1038/s41598-017-11132-0 (PMC5585334; doi:10.1038/s41598-017-11132-0)
Supplement: Supplementary file 1 — Supplementary files [file 41598_2017_11132_MOESM1_ESM.pdf]

Deep-level phylogeny of Cicadomorpha inferred from mitochondrial genomes sequenced by NGS

Nan Song[1], Wanzhi Cai[2], Hu Li[2]

1 College of Plant Protection, Henan Agricultural University, Zhengzhou 450002, China

2 Department of Entomology, China Agricultural University, Beijing 100094, China

Correspondence: Nan Song, College of Plant Protection, Henan Agricultural University, Zhengzhou 450002, China.

E-mail: songnan@henau.edu.cn

Table S1. Taxonomic information and GenBank accession numbers for the taxa included in this study.

Item	Higher Taxon	Superfamily	Family	Species	Accession number	Voucher numbers for the sequenced species
	Cimicomorpha	Membracoidea	Aetalionidae	*Darthula hardwickii*	NC_026699	-
		Membracoidea	Membracidae	*Leptobelus gazella*	NC_023219	-
		Membracoidea	Membracidae	***Tricentrus* sp.**	**KY039115**	EMHAU-15041605
		Membracoidea	Membracidae	***Tricentrus* sp.1**	**KY039118**	EMHAU-15062504
		Membracoidea	Cicadellidae	***Alobaldia tobae***	**KY039116**	EMHAU-15062710
		Membracoidea	Cicadellidae	*Drabescoides nuchalis*	NC_028154	-
		Membracoidea	Cicadellidae	*Empoasca vitis*	NC_024838	-
		Membracoidea	Cicadellidae	***Exitianus indicus***	**KY039128**	EMHAU-15062706
		Membracoidea	Cicadellidae	*Homalodisca vitripennis*	NC_006899	-
		Membracoidea	Cicadellidae	***Illinigina* sp.**	**KY039129**	EMHAU-15062817
		Membracoidea	Cicadellidae	*Nephotettix cincticeps*	NC_026977	-
		Membracoidea	Cicadellidae	***Norvellina* sp.**	**KY039131**	EMHAU-15062816
		Membracoidea	Cicadellidae	***Olidiana* sp.**	**KY039119**	EMHAU-15062716
		Membracoidea	Cicadellidae	***Orosius orientalis***	**KY039146**	EMHAU-15062705
		Membracoidea	Cicadellidae	***Phlogotettix* sp.**	**KY039135**	EMHAU-15090801
		Membracoidea	Cicadellidae	***Typhlocyba* sp.**	**KY039138**	EMHAU-15062510
		Membracoidea	Cicadellidae	***Yanocephalus yanonis***	**KY039113**	EMHAU-15062803
		Cercopoidea	Aphrophoridae	***Peuceptyelus minutus***	**KY039134**	EMHAU-15052918
**Ingroup**		Cercopoidea	Aphrophoridae	*Philaenus spumarius*	AY630340	-
		Cercopoidea	Cercopidae	***Aphrophora* sp.**	**KY039120**	EMHAU-15062701
		Cercopoidea	Cercopidae	*Abidama producta*	NC_015799	-
		Cercopoidea	Cercopidae	***Aeneolamia contigua***	**KY039117**	EMHAU-15070803
		Cercopoidea	Cercopidae	***Aphrophora alni***	**KY039122**	EMHAU-15062722
		Cercopoidea	Cercopidae	*Callitettix versicolor*	EU725832	-
		Cercopoidea	Cercopidae	*Callitettix biformis*	NC_025496	-
		Cercopoidea	Cercopidae	*Callitettix braconoides*	NC_025497	-
		Cercopoidea	Cercopidae	***Callitettix* sp.**	**KY039124**	EMHAU-15090607
		Cercopoidea	Cercopidae	*Cosmoscarta bispecularis*	NC_026289	-

		Cercopoidea	Cercopidae	*Paphnutius ruficeps*	NC 021100	-
		Cicadoidea	Cicadidae	*Diceroprocta semicincta*	KM000131	-
		Cicadoidea	Cicadidae	*Gaeana maculata*	KM244671	-
		Cicadoidea	Cicadidae	*Magicicada tredecim*	KM000130	-
		Cicadoidea	Cicadidae	***Meimuna opalifera***	**KY039112**	EMHAU-16010524
		Cicadoidea	Cicadidae	***Platypleura kaempferi***	**KY039114**	EMHAU-16010602
		Cicadoidea	Cicadidae	***Pomponia linearis***	**KY039136**	EMHAU-16010509
		Cicadoidea	Cicadidae	*Tettigades auropilosa*	KM000129	-
		Cicadoidea	Cicadidae	*Tettigades ulnaria*	KM000128	-
	Fulgoromorpha	Fulgoroidea	Cixiidae	***Oliarus* sp.**	**KY039121**	EMHAU-15053033
		Fulgoroidea	Cixiidae	***Pentastiridius* sp.**	**KY039133**	EMHAU-15062511
		Fulgoroidea	Delphacidae	***Nilaparvata* sp.**	**KY039125**	EMHAU-15062823
		Fulgoroidea	Delphacidae	*Sogatella furcifera*	NC_021417	-
		Fulgoroidea	Derbidae	***Lydda* sp.**	**KY039126**	EMHAU-15091903
		Fulgoroidea	Dictyopharidae	***Scolops* sp.**	**KY039127**	EMHAU-15083115
		Fulgoroidea	Fulgoridae	*Lycorma delicatula*	EU909203	-
**Outgroup**		Fulgoroidea	Issidae	***Sivaloka* sp.**	**KY039137**	EMHAU-15070305
		Fulgoroidea	Issidae	*Sivaloka damnosus*	FJ360694	-
	Heteroptera	Nepoidea	Belostomatidae	*Diplonychus rusticus*	FJ456940	-
		Notonectoidea	Notonectidae	*Enithares tibialis*	FJ456949	-
		Cimicoidea	Coreidae	*Hydaropsis longirostris*	EU427337	-
		Miroidea	Miridae	*Lygus hesperus*	NC_024641	-
		Pentatomoidea	Cydnidae	*Macroscytus gibbulus*	EU427338	-
		Lygaeoidea	Berytidae	*Yemmalysus parallelus*	EU427346	-

Note: The newly sequenced mitogenome sequences are indicated in bold.

Table S2. The information of local blast for bait sequences of each species newly determined in this study.

Species	*cox1*			*cytb*			*rrnS*		
	Score	bits	Identities	Score	bits	Identities	Score	bits	Identities
Tricentrus sp.	1229	619	99%	-	-	-	-	-	-
Tricentrus sp.1	1187	599	100%	-			-	-	-
Alobaldia tobae	1982	1000	99%	1134	572	100%	811	409	95%
Exitianus indicus	1980	999	99%	971	490	96%	835	421	96%
Illinigina sp.	1949	983	99%	1140	575	100%	811	409	97%
Norvellina sp.	-	-	-	-	-	-	906	457	98%
Olidiana sp.	1221	618	100%	1126	568	100%	882	445	98%
Orosius orientalis	1217	614	100%	-	-	-	819	413	96%
Phlogotettix sp.	-	-	-	-	-	-	864	436	96%
Typhlocyba sp.	-	-	-	706	356	91%	353	178	87%
Yanocephalus yanonis	1179	595	99%	-	-	-	775	391	95%
Peuceptyelus minutus	-	-	-	1183	597	100%	-	-	-
Aphrophora sp.	1225	618	100%	-	-	-	-	-	-
Aeneolamia contigua	1978	998	99%	-	-	-	835	421	96%
Aphrophora alni	1941	979	99%	1136	573	100%	85	432	96%
Callitettix sp.	-	-	-	1076	543	98%	406	205	93%
Meimuna opalifera	1233	622	99%	-	-	-	-	-	-
Platypleura kaempferi	1207	609	99%	-	-	-	-	-	-
Pomponia linearis	1243	627	99%	-	-	-	-	-	-
Oliarus sp.	-	-	-	-	-	-	509	257	90%
Pentastiridius sp.	1170	590	100%	-	-	-	-	-	-
Nilaparvata sp.	442	223	83%	182	92	83%	260	131	86%
Lydda sp.	-	-	-	-	-	-	848	428	98%
Scolops sp.	1237	624	99%	-	-	-	-	-	-
Sivaloka sp.	1241	626	99%	-	-	-	876	442	97%

Note: Gaps indicate the failure of PCR amplification or Sanger sequencing.

Table S3. (A) The partition sechemes and best-fitting models selected by PartitionFinder for dataset of 52taxa_PCG.

52taxa_PCG		
**Subset**	**Subset Partitions**	**Best Model**
Partition 1	atp6_cp1, atp8_cp2, cox2_cp1, cytb_cp1, cox3_cp1	GTR+I+G
Partition 2	atp6_cp2, cox2_cp2, cytb_cp2, cox3_cp2	GTR+I+G
Partition 3	atp6_cp3, atp8_cp3, cox2_cp3, cytb_cp3, nad3_cp3, nad6_cp3, cox3_cp3	GTR+G
Partition 4	atp8_cp1, nad2_cp1, nad3_cp1, nad6_cp1	GTR+I+G
Partition 5	cox1_cp1	GTR+I+G
Partition 6	cox1_cp2	GTR+I+G
Partition 7	cox1_cp3	GTR+I+G
Partition 8	nad1_cp1, nad4_cp1, nad4l_cp1, nad5_cp1	GTR+I+G
Partition 9	nad1_cp2, nad4_cp2, nad4l_cp2, nad5_cp2	GTR+I+G
Partition 10	nad1_cp3, nad4_cp3, nad4l_cp3, nad5_cp3	GTR+I+G
Partition 11	nad2_cp2, nad3_cp2, nad6_cp2	GTR+I+G
Partition 12	nad2_cp3	GTR+G

Note: "cp_1" representing the first codon position, "cp_2" representing the second codon position, and "cp_3" representing the third codon position.

Abbreviations used in the Best Model: GTR, General-Time-Reversible model; I, invariant sites; G, discrete Gamma distribution; MTREV, the transition probability matrix of the REV model for mtDNA-encoded proteins; F, the actual frequencies of the protein under study.

Table S3. (B) The partition sechemes and best-fitting models selected by PartitionFinder for dataset of 52taxa_PCG_AA.

52taxa_PCG_AA		
**Subset**	**Subset Partitions**	**Best Model**
Partition 1	atp6, cox2, cox3, cytb	MTREV+I+G+F
Partition 2	atp8, nad2, nad3, nad6	MTREV+I+G+F
Partition 3	cox1	MTREV+I+G+F
Partition 4	nad1, nad4, nad4l, nad5	MTREV+I+G+F

Table S3. (C) The partition sechemes and best-fitting models selected by PartitionFinder for dataset of 52taxa_PCGDegen.

52taxa_PCGDegen		
**Subset**	**Subset Partitions**	**Best Model**
Partition 1	atp6_cp1, cox2_cp1, cytb_cp1, nad3_cp1, cox3_cp1	GTR+I+G
Partition 2	atp6_cp2, cox2_cp2, cytb_cp2, cox3_cp2	GTR+I+G
Partition 3	atp6_cp3, nad2_cp3, nad3_cp3, nad6_cp3	GTR+G
Partition 4	atp8_cp1, atp8_cp3, nad2_cp1, nad6_cp1	GTR+I+G
Partition 5	atp8_cp2, nad1_cp3, nad2_cp2, nad3_cp2, nad6_cp2	GTR+G
Partition 6	cox1_cp1	GTR+G
Partition 7	cox1_cp2	GTR+I+G
Partition 8	cox1_cp3, cox2_cp3, cytb_cp3, cox3_cp3	GTR+G
Partition 9	nad1_cp1, nad4_cp1, nad4l_cp1, nad5_cp1	GTR+I+G
Partition 10	nad1_cp2, nad4_cp2, nad4l_cp2, nad5_cp2	GTR+I+G
Partition 11	nad4_cp3, nad4l_cp3, nad5_cp3	GTR+I+G

Table S3. (D) The partition sechemes and best-fitting models selected by PartitionFinder for dataset of 52taxa_PCGRNA.

Subset	Subset Partitions	Best Model
	**52taxa_PCGRNA**	
**Subset**	**Subset Partitions**	**Best Model**
Partition 1	atp6_cp1, atp8_cp2, cox2_cp1, cytb_cp1, cox3_cp1	GTR+I+G
Partition 2	atp6_cp2, cox2_cp2, cytb_cp2, cox3_cp2	GTR+I+G
Partition 3	atp6_cp3, atp8_cp3, cox2_cp3, cytb_cp3, nad3_cp3, nad6_cp3, cox3_cp3	GTR+G
Partition 4	atp8_cp1, nad2_cp1, nad3_cp1, nad6_cp1	GTR+I+G
Partition 5	cox1_cp1	GTR+I+G
Partition 6	cox1_cp2	GTR+I+G
Partition 7	cox1_cp3	GTR+I+G
Partition 8	Ala, Cys, Ser1, Ser2, nad1_cp1, nad4l_cp1	GTR+I+G
Partition 9	nad1_cp2, nad4_cp2, nad4l_cp2, nad5_cp2	GTR+I+G
Partition 10	nad1_cp3, nad4_cp3, nad4l_cp3, nad5_cp3	GTR+I+G
Partition 11	nad2_cp2, nad3_cp2, nad6_cp2	GTR+I+G
Partition 12	nad2_cp3	GTR+G
Partition 13	nad4_cp1, nad5_cp1	GTR+I+G
Partition 14	12S	GTR+I+G
Partition 15	16S, Val	GTR+I+G
Partition 16	Arg, Asn, Gly, Leu2, Lys, Met, Thr, Trp	GTR+I+G
Partition 17	Asp, Glu, Ile, Leu1	GTR+G
Partition 18	Gln, His, Phe, Pro, Tyr	GTR+I+G

Table S3. (E) The partition sechemes and best-fitting models selected by PartitionFinder for dataset of 52taxa_PCGDegenRNA.

52taxa_PCGDegenRNA		
**Subset**	**Subset Partitions**	**Best Model**
Partition 1	atp6_cp1, cox2_cp1, cytb_cp1, nad3_cp1, cox3_cp1	GTR+I+G
Partition 2	atp6_cp2, cox2_cp2, cytb_cp2, cox3_cp2	GTR+I+G
Partition 3	atp6_cp3, cox1_cp3, cox2_cp3, cytb_cp3, nad3_cp3, cox3_cp3	GTR+G
Partition 4	atp8_cp1, atp8_cp3, nad2_cp1, nad6_cp1	GTR+I+G
Partition 5	Ala, Cys, Val, atp8_cp2	GTR+I+G
Partition 6	cox1_cp1	GTR+I+G
Partition 7	cox1_cp2	GTR+I+G
Partition 8	nad1_cp1, nad4_cp1, nad4l_cp1, nad5_cp1	GTR+I+G
Partition 9	nad1_cp2, nad4_cp2, nad4l_cp2, nad5_cp2	GTR+I+G
Partition 10	nad1_cp3, nad2_cp2, nad3_cp2, nad6_cp2	GTR+G
Partition 11	nad2_cp3, nad6_cp3	GTR+G
Partition 12	nad4_cp3, nad4l_cp3, nad5_cp3	GTR+I+G
Partition 13	12S, 16S	GTR+I+G
Partition 14	Arg, Asn, Ile, Lys, Met, Ser1, Ser2	GTR+I+G
Partition 15	Asp, Glu, Gly, Leu1, Leu2, Thr, Trp	GTR+G
Partition 16	Gln, His, Phe, Pro, Tyr	GTR+I+G

Table S3. (F) The partition sechemes and best-fitting models selected by PartitionFinder for dataset of 43taxa_PCG.

	43taxa_PCG	
**Subset**	**Subset Partitions**	**Best Model**
Partition 1	atp6_cp1, atp8_cp2, cox2_cp1, cytb_cp1, cox3_cp1	GTR+I+G
Partition 2	atp6_cp2, cox2_cp2, cytb_cp2, cox3_cp2	GTR+I+G
Partition 3	atp6_cp3, atp8_cp3, cox2_cp3, cytb_cp3, nad3_cp3, nad6_cp3, cox3_cp3	GTR+G
Partition 4	atp8_cp1, nad2_cp1, nad3_cp1, nad6_cp1	GTR+I+G
Partition 5	cox1_cp1	GTR+I+G
Partition 6	cox1_cp2	GTR+I+G
Partition 7	cox1_cp3	GTR+I+G
Partition 8	nad1_cp1, nad4_cp1, nad4l_cp1, nad5_cp1	GTR+I+G
Partition 9	nad1_cp2, nad4_cp2, nad4l_cp2, nad5_cp2	GTR+I+G
Partition 10	nad1_cp3, nad4_cp3, nad4l_cp3, nad5_cp3	GTR+I+G
Partition 11	nad2_cp2, nad3_cp2, nad6_cp2	GTR+I+G
Partition 12	nad2_cp3	GTR+G

Table S3. (G) The partition sechemes and best-fitting models selected by PartitionFinder for dataset of 43taxa_PCG_AA.

43taxa_PCG_AA		
**Subset**	**Subset Partitions**	**Best Model**
Partition 1	atp6, cox2, cox3, cytb	MTREV+I+G+F
Partition 2	atp8, nad2, nad3, nad6	MTREV+I+G+F
Partition 3	cox1	MTREV+I+G+F
Partition 4	nad1, nad4, nad4l, nad5	MTREV+I+G+F

Table S3. (H) The partition sechemes and best-fitting models selected by PartitionFinder for dataset of 43taxa_PCGDegen.

43taxa_PCGDegen		
**Subset**	**Subset Partitions**	**Best Model**
Partition 1	atp6_cp1, cox2_cp1, cytb_cp1, nad3_cp1, cox3_cp1	GTR+I+G
Partition 2	atp6_cp2, cox2_cp2, cytb_cp2, cox3_cp2	GTR+I+G
Partition 3	atp6_cp3, atp8_cp2, nad3_cp3	GTR+G
Partition 4	atp8_cp1, atp8_cp3, nad2_cp1, nad6_cp1	GTR+I+G
Partition 5	cox1_cp1	GTR+I+G
Partition 6	cox1_cp2	GTR+I+G
Partition 7	cox1_cp3, cox2_cp3, cytb_cp3, cox3_cp3	GTR+I+G
Partition 8	nad1_cp1	GTR+G
Partition 9	nad1_cp2, nad4_cp2, nad4l_cp2, nad5_cp2	GTR+I+G
Partition 10	nad1_cp3, nad4_cp3, nad4l_cp3, nad5_cp3	GTR+G
Partition 11	nad2_cp2, nad3_cp2, nad6_cp2	GTR+I+G
Partition 12	nad2_cp3, nad6_cp3	GTR+G
Partition 13	nad4_cp1, nad4l_cp1, nad5_cp1	GTR+I+G

Table S3. (I) The partition sechemes and best-fitting models selected by PartitionFinder for dataset of 43taxa_PCGRNA.

43taxa_PCGRNA		
**Subset**	**Subset Partitions**	**Best Model**
Partition 1	Ala, Cys, Gln, Val, atp6_cp1, atp8_cp2	GTR+I+G
Partition 2	atp6_cp2, cox2_cp2, cytb_cp2, cox3_cp2	GTR+I+G
Partition 3	atp6_cp3, atp8_cp3, cox2_cp3, cytb_cp3, nad3_cp3, nad6_cp3, cox3_cp3	GTR+G
Partition 4	atp8_cp1, nad2_cp1, nad3_cp1, nad6_cp1	GTR+I+G
Partition 5	cox1_cp1	GTR+I+G
Partition 6	cox1_cp2	GTR+I+G
Partition 7	cox1_cp3	GTR+I+G
Partition 8	cox2_cp1, cytb_cp1, cox3_cp1	GTR+I+G
Partition 9	nad1_cp1, nad4_cp1, nad4l_cp1, nad5_cp1	GTR+I+G
Partition 10	nad1_cp2, nad4_cp2, nad4l_cp2, nad5_cp2	GTR+I+G
Partition 11	nad1_cp3, nad4_cp3, nad4l_cp3, nad5_cp3	GTR+I+G
Partition 12	nad2_cp2, nad3_cp2, nad6_cp2	GTR+I+G
Partition 13	nad2_cp3	GTR+G
Partition 14	12S, 16S	GTR+I+G
Partition 15	Arg, Asn, Gly, Ile, Lys, Met, Ser1, Ser2, Thr, Trp	GTR+I+G
Partition 16	Asp, Glu, His, Leu1, Leu2, Phe, Pro, Tyr	GTR+I+G

Table S3. (J) The partition sechemes and best-fitting models selected by PartitionFinder for dataset of 43taxa_PCGDegenRNA.

43taxa_PCGDegenRNA		
**Subset**	**Subset Partitions**	**Best Model**
Partition 1	atp6_cp1, cox2_cp1, cytb_cp1, nad3_cp1, cox3_cp1	GTR+I+G
Partition 2	atp6_cp2, cox2_cp2, cytb_cp2, cox3_cp2	GTR+I+G
Partition 3	atp6_cp3, cox2_cp3, cytb_cp3, nad1_cp3, nad4_cp3, nad4l_cp3, nad5_cp3, cox3_cp3	GTR+I+G
Partition 4	atp8_cp1, atp8_cp3, nad2_cp1, nad6_cp1	GTR+I+G
Partition 5	atp8_cp2, nad2_cp2, nad3_cp2, nad3_cp3, nad6_cp2	GTR+G
Partition 6	cox1_cp1	GTR+G
Partition 7	cox1_cp2	GTR+I+G
Partition 8	cox1_cp3, nad1_cp2	GTR+G
Partition 9	nad1_cp1, nad4_cp1, nad4l_cp1, nad5_cp1	GTR+I+G
Partition 10	nad2_cp3, nad6_cp3	GTR+G
Partition 11	nad4_cp2, nad4l_cp2, nad5_cp2	GTR+I+G
Partition 12	12S, 16S, Val	GTR+I+G
Partition 13	Ala, Cys, Gln, Ser1, Ser2	GTR+I+G
Partition 14	Arg, Asn, Asp, Glu, Gly, His, Ile, Leu1, Leu2, Lys, Met, Phe, Pro, Thr, Trp, Tyr	GTR+I+G
